# Clinical impact of guideline-based practice and patients’ adherence in uncontrolled hypertension

**DOI:** 10.1186/s40885-021-00183-1

**Published:** 2021-12-15

**Authors:** Il Suk Sohn, Chong Jin Kim, Byung-Su Yoo, Byung Jin Kim, Jae Woong Choi, Doo-Il Kim, Sang-Hak Lee, Woo-Hyuk Song, Dong Woon Jeon, Tae Jun Cha, Dae-Kyeong Kim, Seong-Hoon Lim, Chang-Wook Nam, Joon-Han Shin, Ung Kim, Jae-Jin Kwak, Jun-Bean Park, Jin-Hye Cha, Young-Joo Kim, Jimi Choi, Juneyoung Lee

**Affiliations:** 1 Kyung Hee University Hospital at Gangdong, Seoul, Republic of Korea; 2 Department of Cardiology, CHA University Gangnam Medical Center, Seoul, Republic of Korea; 3 Wonju Severance Christian Hospital, Yonsei University Health System, Wonju, Republic of Korea; 4 Kangbuk Samsung Hospital, Sungkyunkwan University School of Medicine, Seoul, Republic of Korea; 5 Eulji General Hospital, Seoul, Republic of Korea; 6 Inje University Haeundae Paik Hospital, Busan, Republic of Korea; 7 Severance Hospital, Yonsei University College of Medicine, Seoul, Republic of Korea; 8 Korea University Ansan Hospital, Ansan, Republic of Korea; 9 National Health Insurance Service Ilsan Hospital, Goyang, Republic of Korea; 10 Kosin University Gospel Hospital, Busan, Republic of Korea; 11 Inje University Busan Paik Hospital, Busan, Republic of Korea; 12 Dankook University Hospital, Cheonan, Republic of Korea; 13 Keimyung University Dongsan Hospital, Daegu, Republic of Korea; 14 Ajou University Hospital, Suwon, Republic of Korea; 15 Yeungnam University Hospital, Daegu, Republic of Korea; 16 Inje University Ilsan Paik Hospital, Goyang, Republic of Korea; 17 Seoul National University Hospital, Seoul, Republic of Korea; 18 Viatris Korea Ltd., Seoul, Republic of Korea; 19 Pfizer Korea, Seoul, Republic of Korea; 20 Korea University College of Medicine, Seoul, Republic of Korea

**Keywords:** Treatment adherence and compliance, Quality of life, Patient satisfaction

## Abstract

**Background:**

Chronic diseases like hypertension need comprehensive lifetime management. This study assessed clinical and patient-reported outcomes and compared them by treatment patterns and adherence at 6 months among uncontrolled hypertensive patients in Korea.

**Methods:**

This prospective, observational study was conducted at 16 major hospitals where uncontrolled hypertensive patients receiving anti-hypertension medications (systolic blood pressure ≥ 140 mmHg or diastolic blood pressure ≥ 90 mmHg) were enrolled during 2015 to 2016 and studied for the following 6 months. A review of medical records was performed to collect data on treatment patterns to determine the presence of guideline-based practice (GBP). GBP was defined as: (1) maximize first medication before adding second or (2) add second medication before reaching maximum dose of first medication. Patient self-administered questionnaires were utilized to examine medication adherence, treatment satisfaction and quality of life (QoL).

**Results:**

A total of 600 patients were included in the study. Overall, 23% of patients were treated based on GBP at 3 months, and the GBP rate increased to 61.4% at 6 months. At baseline and 6 months, 36.7 and 49.2% of patients, respectively, were medication adherent. The proportion of blood pressure-controlled patients reached 65.5% at 6 months. A higher blood pressure control rate was present in patients who were on GBP and also showed adherence than those on GBP, but not adherent, or non-GBP patients (76.8% vs. 70.9% vs. 54.2%, *P* < 0.001). The same outcomes were found for treatment satisfaction and QoL (*P* < 0.05).

**Conclusions:**

This study demonstrated the importance of physicians’ compliance with GBP and patients’ adherence to hypertensive medications. GBP compliance and medication adherence should be taken into account when setting therapeutic strategies for better outcomes in uncontrolled hypertensive patients.

**Supplementary Information:**

The online version contains supplementary material available at 10.1186/s40885-021-00183-1.

## Background

Hypertension is one of the major causes of death and the leading risk factor for cardiovascular disease and mortality worldwide [1]. However, achieving and maintaining blood pressure (BP) goals in hypertension has been challenging. About one-third of hypertensive patients are unaware of this condition or, if aware, do not undergo treatment, and target BP values are seldom achieved. This failure to control BP is associated with persistent elevated cardiovascular risk [2]. Most guidelines are based on evidence from multiple randomized clinical trials (RCTs) and recommend that the clinician should continue to assess BP and adjust the treatment regimen until goal BP is reached. If goal BP is not reached, guidelines recommend increasing the dose of the initial drug or adding a second drug from one of the recommended classes [2–6].

In real-world practice, most clinicians often care for patients with numerous comorbidities or other challenging issues, making BP control more difficult and this may be one of the reasons that clinicians do not follow guideline-based practice (GBP). In addition, almost half of patients discontinue treatment leading to poor BP control [7]. Poor adherence to medication can lead to cardiovascular morbidity and mortality [8, 9]. It has been established that medication adherence and BP control to recommended goal lead to a decrease in hypertension-related morbidity and mortality in hypertensive patients resulting in satisfaction with care and improvement in health-related quality of life (QoL) [10–12].

This study aimed to assess treatment patterns and medication adherence and to compare clinical (BP control) and patient-reported outcomes (treatment satisfaction and QoL) by treatment patterns and medication adherence at 6 months among uncontrolled hypertensive patients.

## Methods

### Patients and study design

A non-interventional, prospective and observational study was conducted at 16 nationwide, tertiary hospitals. Study patients were enrolled during 2015 to 2016 and assessed for the following 6 months. Eligible patients were aged over 20 years with uncontrolled hypertension, determined by 2 to 3 repeated clinic BP measurements (systolic BP ≥140 mm/Hg or diastolic BP ≥90 mmHg) at the time of enrolment. Patients with resistant hypertension, secondary causes of hypertension, or those enrolled in another drug intervention study, were excluded. The total study period for each enrolled patient was 6 months and patients were assessed at their regular visit at 3 months and 6 months after receiving antihypertensive medications.

Data were collected through a review of medical records and face-to-face patient interviews. Demographic data included age, gender, smoking status, alcohol behavior, regular exercise, lipid lowering diet, and education level. BP was measured by the attending physician using a standardized protocol with a validated mercury sphygmomanometer and an appropriate cuff size for the arm circumference. Researchers reviewed electronic medical records for asymptomatic organ damage (albuminuria, left ventricular hypertrophy on electrocardiogram, retinopathy, and arterial stiffening) and hypertension-related underlying disease (renal disease, cerebrovascular disease, diabetes, peripheral arterial disease, heart failure, and coronary artery disease). Physicians prescribed antihypertensive medications at their discretion without the need to follow any regulations or protocols at each patient’s visit. Treatment patterns were used to examine whether physicians followed GBP, which was based on the Joint National Committee 8 guideline [6] and was defined if one of following criteria was met; (1) maximize the first medication before adding a second or (2) add a second medication before reaching the maximum dose of the first medication to control BP. All subjects gave informed consent and the study was conducted after approval from the institutional review board at each hospital.

### Assessment of medication adherence and patient-reported outcomes

Among the various methods of assessing medication adherence, we evaluated adherence using the 8-item Morisky Medication Adherence Scale (MMAS-8) with three levels of adherence (high, medium, low) [13–15]. The Korean version of the MMAS-8 was used for data collection and licensure agreement with the survey provider, Donald E. Morisky (dmorisky@gmail.com), was obtained. After approval for its use, treatment satisfaction was assessed using the Korean version of the Treatment Satisfaction Questionnaire for Medication, version 1.4 (TSQM 1.4), consisting of four domains (effectiveness, side effects, convenience, global satisfaction) [16]. TSQM 1.4 domain scores range from 0 to 100, with higher scores representing higher satisfaction in three of the domains (effectiveness, side effects, convenience) regarding patients’ antihypertensive medications. The “global satisfaction” domain was used to assess the overall level of satisfaction or dissatisfaction with medications. The Korean version of the EuroQoL-visual analog scale (EQ-VAS, Rotterdam, Netherlands) was used (with permission) to evaluate patient QoL regarding antihypertensive treatment. Patients were asked to indicate how good or bad their health state is and check the point on the scale numbered from 0 (worst) to 100 (best). MMAS-8 was assessed three times, at the recruitment visit and at both follow-up visits; TSQM 1.4 and EQ-VAS were assessed at the recruitment visit and at the end-of-study visit. Patients were categorized as (1) GBP and adherent group, (2) GBP and non-adherent group, and (3) non-GBP according to GBP status and medication adherent by MMAS-8.

### Statistical analysis

This study compared clinical and patient-reported outcomes between GBP and non-GBP groups, and between adherent and non-adherent groups. For the description of patients’ characteristics, continuous variables were presented with basic statistics (the number of observations, means and standard deviations), whereas frequency and percentage (%) were reported for categorical variables. For two-group comparisons, the generalized estimating equation (GEE) method was performed to compare the rates of GBP and adherence and the BP control rate, at different observation periods. Likewise, the paired t-test was used to estimate differences in treatment satisfaction and QoL between baseline and 6-month follow-up. Three group comparisons were conducted with the chi-square test for BP control and with ANOVA and/or Kruskal-Wallis test for treatment satisfaction and QoL. Only patients who visited at each observational period and completed the survey were included in group comparisons. A multivariable logistic regression analysis was conducted for BP control while multivariable linear regression analyses were applied to treatment satisfaction and QoL. For the multivariable analyses, factors that were found to be present from univariate analysis with a significance level of 10% (*P* < 0.1), and clinically meaningful, were adjusted. SAS ver. 9.4 (SAS Institute Inc., Cary, NC, USA) was used for all statistical analyses.

## Results

### Study subjects

Table 1 explains baseline characteristics of the study subjects. This study included a total of 600 uncontrolled hypertensive patients (mean age 58.6 ± 13.4 years, 55.7% male) (Table 1). The mean duration of hypertension from the first diagnosis was 7.4 ± 6.7 years and the mean duration of treatment for hypertension was 6.8 ± 6.7 years. One hundred and fifty patients (25%) had asymptomatic organ damage and 113 patients (18.8%) had hypertension-related underlying diseases (Table 1). Patient characteristics between GBP and non-GBP, and between adherent and non-adherent groups at 6 months are described in the Table S1.

**Table 1:** Patient characteristics at baseline (*n* = 600)

Characteristic	Variable
Male sex	334 (55.7)
Age (yr)	58.6 ± 13.4
Body mass index (kg/m^2^)	25.6 ± 3.7
Education	
No	27 (4.5)
≤ High school graduation	313 (52.2)
≥ College graduation	255 (42.5)
Unknown	5 (0.8)
Smoking	
Non-smoker	364 (60.7)
Ex-smoker	135 (22.5)
Current smoker	99 (16.5)
Unknown	2 (0.3)
Alcohol consumption	
Non-drinker	235 (39.2)
Ex-drinker	55 (9.2)
Current drinker	309 (51.5)
Unknown	1 (0.1)
Exercise^a^ (times/wk)	
≤ 2	316 (52.7)
≥ 3	284 (47.3)
Lipid lowering diet	267 (44.5)
Duration of hypertension (yr)	7.4 ± 6.7
Duration of treatment of hypertension (yr)	6.8 ± 6.7
Asymptomatic organ damage^b^	150 (25.0)
Hypertension-related underlying disease^c^	113 (18.8)

Data are presented as number (%) or mean ± standard deviation

^a^Repeated exercises that patients spent more than 30 min per time, and it examined on weekly basis

^b^Asymptomatic organ damage includes albuminuria, left ventricular hypertrophy on electrocardiogram, retinopathy, and arterial stiffening

^c^Hypertension-related underlying diseases are renal disease, cerebrovascular disease, diabetes, peripheral arterial disease, heart failure, or coronary artery disease

### Guideline-based practice, medication adherence and blood pressure control

**Fig. 1:**
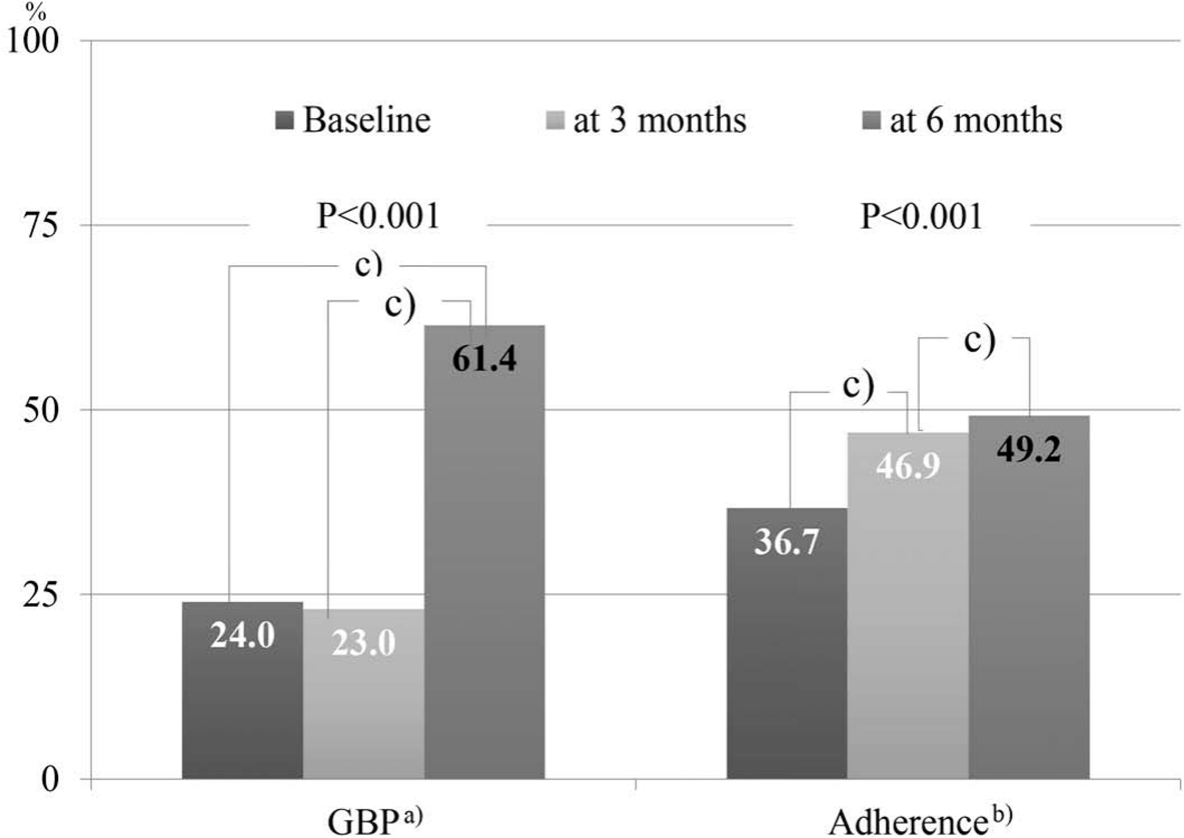
Guideline-based practice and medication adherence. ^a)^Guideline-based practice (GBP) was based on the JNC 8 guidelines and defined as systolic BP (SBP) ≥140 mmHg or diastolic BP (DBP) ≥90 mmHg and with the treatment strategies for antihypertensive drugs meeting one of the following: (1) maximize first medication before adding second, (2) add second medication before reaching the maximum dose of the first medication, or (3) start with two medication classes separately or as a fixed-dose combination. If BP was controlled (either SBP < 140 mmHg or DBP < 90 mmHg) at the next visit, GBP was determined as the same treatment strategies were implemented. ^b)^ Adherence was defined as patients showing high adherence according to Morisky Medication Adherence Scale-8 and moderate and low adherent patients were categorized as non-adherent [13–15]. ^c)^ Indicates that a comparison of 2 values showed a significant difference at *P* < 0.001. *P*-values for visit effect by generalized estimating equation method

Overall, 23% of patients were treated based on GBP at 3 months, and the GBP rate increased to 61.4% at 6 months (*P* < 0.001 by GEE method) (Fig. 1). The percentage of adherent patients was 36.7% at baseline, increasing to 49.2% at 6 months (*P <* 0.001 by mixed model for repeated measurements). The proportion of BP-controlled patients increased during the study period, reaching 65.5% at 6 months (Table 2). In a multivariate analysis, BP control rate in the GBP and adherent group (odds ratio [OR] 2.65, 95% confidence interval [CI] 1.58-4.42) and the GBP and non-adherent group (OR 1.67, 95% CI 1.02-2.76) was higher than in the non-GBP group (Table 3).

**Table 2:** BP and control rate

Variable	At baseline (*n* = 600)		At 3 mo (*n* = 436)		At 6 mo (*n* = 502)		*P*-value
	n	Mean ± SD	n	Mean ± SD	n	Mean ± SD	
SBP (mmHg)	600	148.5 ± 13.1	430	132.6 ± 14.9	493	133.5 ± 14.6	< 0.001^a^
DBP (mmHg)	600	89.7 ± 10.5	430	79.5 ± 10.6	493	79.8 ± 10.7	< 0.001 ^a^
BP control rate (%)^b^	600	0	430	64.2	493	65.5	< 0.001^c^

*SD* standard deviation SBP, systolic blood pressure, *DBP* diastolic blood pressure

^a^*P*-value for visit effect by mixed model for repeated measurement

^b^SBP < 140 mmHg and DBP < 90 mmHg at each observation period

^c^*P*-value for visit effect by binary logistic regression using generalized estimation equations method

Among the total number of patients at each observation, those with no data on BP were excluded in the analysis

**Table 3:** Multivariable analysis for blood pressure control according to GBP and adherence status (*n* = 457)

GBP and adherence status	Odds ratio^a^	95% CI	*P*-value
Non-GBP (*n* = 178)	Ref.	Ref.	
GBP and adherence (*n* = 140)	2.65	1.58–4.42	< 0.001
GBP and non-adherence (*n* = 139)	1.67	1.02–2.76	0.043

*GBP* guideline-based practice, *CI* confidence interval

^a^Adjusted factors included age, sex, and other variables, body mass index, education, dyslipidaemia diagnosis, asymptomatic organ damage, and underlying disease related to hypertension, which were found to be significant at *P <* 0.1 from univariate analysis

### Guideline-based practice, medication adherence and patient-reported outcomes

Better treatment satisfaction was observed in the GBP and adherent group compared with the GBP and non-adherent, or non-GBP patients, in all domains (all *P* < 0.05) (Table 4). Patients who were treated according to GBP and adherent to their antihypertensive medications had better QoL than in both other groups of patients (*P* = 0.030) (Table 4).

**Table 4:** Patients-reported outcomes according to the status on GBP and medication adherence at 6 months

Variable	GBP				Non-GBP (*n* = 182)		Total (*n* = 502)		*P*-value^*^
	Adherence (*n* = 142)		Non-adherence (*n* = 146)						
	n	Mean ± SD	n	Mean ± SD	n	Mean ± SD	n	Mean ± SD	
TSQM domains									
Effectiveness	141	66.5 ± 12.0^b^	146	62.4 ± 13.5^c^	181	61.4 ± 12.7^c^	500	63.2 ± 12.8	0.001
Side effects	140	99.5 ± 4.2^b^	146	97.6 ± 10.4^c^	181	99.2 ± 5.9^b^	499	98.8 ± 7.2	0.048
Convenience	141	72.1 ± 10.1^b^	146	64.1 ± 12.7^c^	181	65.4 ± 11.7^c^	500	66.9 ± 12.0	< 0.001
Global satisfaction	141	65.7 ± 12.1^b^	146	60.5 ± 14.2^c^	181	59.2 ± 13.7^c^	500	61.2 ± 13.7	< 0.001
EQ-VAS	140	77.6 ± 11.7^b^	146	73.8 ± 14.7^c^	181	74.4 ± 13.1^b,c^	499	74.8 ± 13.7	0.030

*GBP* guideline-based practice, *TSQM* Treatment Satisfaction Questionnaire for Medication, *EQ-VAS* EuroQoL-visual analog scale

^*^All *P-*values among three comparing groups by ANOVA

^b, c^There is a significant difference between b and c by a post hoc Tukey’s test

Among the total number of patients in each group, those with no data on TSQM or EQ-VAS were excluded in the analysis

## Discussion

This multicenter, prospective, observational study demonstrated that physicians’ compliance with GBP and patients’ good adherence to prescribed medications are important to improve BP control, treatment satisfaction, and QoL. Overall, the GBP rate increased during the 6-month study period. Physicians tended to follow GBP throughout the study period and more patients showed better adherence at the end-of-study visit than at baseline (36.7% vs. 49.2%). Both physicians’ and patients’ good compliance to the treatment of hypertension led to an increase in BP control at 6 months in patients with uncontrolled hypertension. The non-GBP group, in which physicians did not follow GBP, showed the lowest BP control rate at 6 months and this was even lower than in the GBP but non-adherent group (70.9% vs. 54.2%). This result highlights the unfavorable effects of physician inertia (i.e., lack of therapeutic action when the patient’s BP is not controlled) in the treatment of hypertension in real-world practice [2]. In addition to this physician inertia, poor adherence to medication is the most important cause of poor BP control [17, 18]. After 6 months and 1 year, more than one-third and about one-half of patients, respectively, may stop their initial treatment [19]. In our non-interventional, observational study, adherence increased from about one-third of patients at baseline up to almost one-half of all patients at 6 months, mainly due to the GBP effect.

Patients’ satisfaction with their treatment is highly associated with compliant medication use, thereby affecting the clinical effectiveness and efficiency of medical care. Treated hypertensive patients with low treatment satisfaction may be more likely to have lower adherence to antihypertensive medications. Low satisfaction with treatment may be an important barrier to achieving high rates of treatment adherence [11]. There are several ways to assess patients’ satisfaction with their treatment. In the current study, we used TSQM which provides information to compare various medications used to treat a particular illness on the three primary dimensions of treatment satisfaction (effectiveness, side effects, convenience), as well as patients’ overall rating of global satisfaction based on the relative importance of these primary dimensions to patients [16]. We found that patients who were treated according to GBP and also adherent to their antihypertensive medications (GBP and adherent group) not only had a higher BP control rate, but also higher satisfaction with their treatment and better QoL than the other two groups of patients in the study.

In real life, poor adherence to antihypertensive medication leads to cardiovascular events and mortality [8, 9, 20]. In other words, poor adherence to antihypertensive therapy correlates with a higher risk of cardiovascular events [19, 20]. In contrast, it has been shown that good adherence to antihypertensive medications has positive impacts on clinical and patient-reported outcomes including treatment satisfaction and QoL [10–12]. Based on evidence from multiple RCTs, recent hypertension guidelines and meta-analyses recommend more intensive BP control in adult hypertensive patients to reduce the risk of cardiovascular disease and all-cause mortality [21–23]. To improve outcomes for hypertensive patients, physicians are recommended to make every effort to follow GBP and to improve adherence to antihypertensive treatment and BP control. However, the majority of treated hypertensive patients are unlikely to achieve recommended BP targets in real life.

Despite the meaningful findings in real-world healthcare settings, this study has a couple of limitations. First, caution is needed regarding the generalizability of the study results since the study only involved major tertiary hospitals which inevitably excluded patients who usually visit local clinics. Therefore, a study including various types of hospitals needs to be conducted for more clarification. Second, there might have been reporting bias resulting from recall bias of the responders regarding the nature of data collection. Measuring medication adherence was based solely on patients’ self-report which may have mistakenly underestimated or overestimated adherence. Objective methodology for the assessment of medication adherence may more clearly explain actual adherence levels in hypertensive patients.

## Conclusions

This study demonstrated the importance of physicians’ compliance with GBP and patients’ adherence to prescribed antihypertensive medications to improve BP control, treatment satisfaction, and QoL. GBP compliance and medication adherence should be taken into account when setting therapeutic strategies in order to lead to better outcomes in patients with hypertension.

## Supplementary Information

**Additional file 1: Table S1.** Patient characteristics at 6 months.

## Data Availability

All data generated or analysed during this study are included in this published article.

## Abbreviations

BP: Blood pressure
CI: Confidence interval
EQ-VAS: EuroQoL-visual analog scale
GBP: Guideline-based practice
GEE: Generalized estimating equation
MMAS-8: 8-item Morisky Medication Adherence Scale
OR: Odds ratio
QoL: Quality of life
RCTs: Randomized clinical trials
SD: Standard deviation
TSQM 1.4: Treatment Satisfaction Questionnaire for Medication, version 1.4
