# Supplementary material for: Clinical impact of guideline-based practice and patients’ adherence in uncontrolled hypertension
Source: Clin Hypertens. 2021 Dec 15;27:26. doi: 10.1186/s40885-021-00183-1 (PMC8672596; doi:10.1186/s40885-021-00183-1)
Supplement: Supplementary file 1 — Additional file 1: Table S1. Patient characteristics at 6 months. [file 40885_2021_183_MOESM1_ESM.docx]

**Table S1.** Patient characteristics at 6 months

| Characteristic | Total^a)^  (n = 470) | GBP  (n = 288) | Non-GBP  (n = 182) | Adherence  (n = 233) | Non-adherence  (n = 237) |
| --- | --- | --- | --- | --- | --- |
| Male sex | 254 (54.0) | 155 (53.8) | 99 (54.4) | 132 (56.7) | 122 (51.5) |
| Age (yr) | 59.7 ± 12.7 | 60.3 ± 12.7 | 58.7 ± 12.7 | 60.5 ± 12.6 | 58.9 ± 12.8 |
| Body mass index (kg/m^2^) | 25.6 ± 3.6 | 25.4 ± 3.6 | 25.9 ± 3.5 | 25.5 ± 3.5 | 25.7 ± 3.7 |
| Education |  |  |  |  |  |
| No | 23 (5.0) | 11 (3.8) | 12 (6.6) | 14 (6.0) | 9 (3.8) |
| ≤ High school graduation | 257 (54.7) | 162 (56.3) | 95 (52.2) | 133 (57.1) | 124 (52.3) |
| ≥ College graduation | 185 (39.4) | 111 (38.5) | 74 (40.7) | 84 (36.0) | 101 (42.6) |
| Unknown | 5 (1.1) | 4 (1.4) | 1 (0.6) | 2 (0.9) | 3 (1.3) |
| Smoking |  |  |  |  |  |
| Non-smoker | 292 (62.1) | 182 (63.2) | 110 (60.4) | 145 (62.2) | 147 (62.0) |
| Ex-smoker | 107 (22.8) | 62 (21.5) | 45 (24.7) | 57 (24.5) | 50 (21.1) |
| Current smoker | 69 (14.7) | 42 (14.6) | 27 (14.8) | 30 (12.9) | 39 (16.5) |
| Unknown | 2 (0.4) | 2 (0.7) | 0 | 1 (0.4) | 1 (0.4) |
| Alcohol consumption |  |  |  |  |  |
| Non-drinker | 196 (41.7) | 118 (41.0) | 78 (42.9) | 93 (39.9) | 103 (43.5) |
| Ex-drinker | 44 (9.4) | 30 (10.4) | 14 (7.7) | 20 (8.6) | 24 (10.1) |
| Current drinker | 229 (48.7) | 140 (48.6) | 89 (48.9) | 120 (51.5) | 109 (46.0) |
| Unknown | 1 (0.2) | 0 (0) | 1 (0.6) | 0 (0) | 1 (0.4) |
| Exercise^b)^ (times/wk) |  |  |  |  |  |
| ≤ 2 | 249 (53.0) | 103 (56.6) | 146 (50.7) | 121 (51.9) | 128 (54.0) |
| ≥ 3 | 221 (47.0) | 79 (43.4) | 142 (49.3) | 112 (48.1) | 109 (46.0) |
| Lipid lowering diet | 213 (45.3) | 140 (48.6) | 73 (40.1) | 119 (51.1) | 94 (39.7) |
| Duration of hypertension (yr) | 7.8 ± 6.3 | 7.5 ± 6.0 | 8.3 ± 6.7 | 7.8 ± 6.1 | 7.8 ± 6.5 |
| Duration of treatment of hypertension (yr) | 7.1 ± 6.3 | 6.9 ± 5.9 | 7.6 ± 6.9 | 7.3 ± 6.1 | 7.0 ± 6.5 |
| Asymptomatic organ damage^c)^ | 132 (28.1) | 94 (32.6) | 38 (20.9) | 53 (22.8) | 79 (33.3) |
| Hypertension-related underlying disease^d)^ | 98 (20.9) | 57 (19.8) | 41 (22.5) | 49 (21.0) | 49 (20.7) |

Data are presented as number (%) or mean ± standard deviation.

GBP, guideline-based practice.

^a)^Patients whose data were available for the assessment of on GBP and adherence at 6 months were only included. ^b)^Repeated exercises that patients spent more than 30 minutes per time, and it examined on weekly basis. ^c)^Asymptomatic organ damage includes albuminuria, left ventricular hypertrophy on electrocardiogram, retinopathy, and arterial stiffening. ^d)^Hypertension-related underlying diseases are renal disease, cerebrovascular disease, diabetes, peripheral arterial disease, heart failure, or coronary artery disease.
